# Effects of silver diammine fluoride with/without potassium iodide on enamel and dentin carious lesions in primary teeth

**DOI:** 10.3389/froh.2024.1465956

**Published:** 2024-08-26

**Authors:** M. Kaur, P. Anderson, S. Shahid, G. R. Davis, D. Mills, F. S. L. Wong

**Affiliations:** Centre for Oral Bioengineering, Barts and the London School of Medicine and Dentistry Queen Mary, University of London, London, United Kingdom

**Keywords:** SDF, KI, remineralization, XMT, SEM

## Abstract

**Aim:**

To assess the effects of SDF and SDF+KI treatment on enamel and dentin carious lesions in primary teeth using x-ray Microtomography (XMT) and back scattered scanning electron microscopy (BSE-SEM).

**Methods:**

Artificial enamel caries of 3 caries free primary teeth were created by immersion of the samples in 50 ml demineralization solution for 72 h. Three other teeth with natural dentin caries were selected. Both groups were divided into 3 subgroups: EC–Enamel Control; ES–Enamel with SDF application; ESK–Enamel with SDF followed by KI application; DC–Dentin Control; DS–Dentin with SDF application; DSK–Dentin with SDF followed by KI application. Each tooth was imaged using XMT at 3 time points: (1) Pretreatment; (2) after immersion in remineralization solution for 120 h, with or without SDF or SDF+KI; (3) after subsequent immersion in demineralization solution for 72 h. The change of radiopacities of the lesions in these time points were assessed from the XMT images. After the XMT scans, all teeth were investigated microscopically using BSE-SEM.

**Results:**

In EC, no change in linear attenuation coefficient (LAC) was observed after remineralization, but LAC reduction was observed after subsequent demineralization. For ES, thin layer of high LAC material was deposited on the enamel surface after remineralization, and further reduction of LAC was observed after demineralization. In ESK, the surface layer was lost after SDF+KI, and small reduction of LAC was observed after demineralization. In DC, no LAC change was observed after remineralization, but reduction of LAC was detected after demineralization. In DS, high LAC material was formed on the carious dentin surface and randomly inside the lesion. No further LAC change was found after demineralization. In DSK, thick layer of high LAC material was deposited on the carious surface and inside the dentinal tubules. No further LAC reduction was found after subsequent demineralization.

**Conclusion:**

SDF and SDF+KI did not protect artificial enamel under acid attack even though Ag products were deposited in the porous enamel. However, SDF and SDF+KI shows protective properties against acid challenges and Ag products are deposited in carious dentin lesion without tubular structure randomly; and within dentinal tubules when these structures are retained.

## 1 Introduction

The application of Silver Diammine Fluoride (SDF) on carious dentin lesion has been shown to arrest caries progression by forming a black remineralized surface layer (1–3). This black discoloration is caused by metallic silver (Ag) which is a photo-reduced product from silver chloride (AgCl), a compound formed by SDF reacting with saliva (4). Riva Star (SDI, Australia), a commercial SDF brand, has an optional additional application of potassium iodide (KI) solution to remove the discolouration by binding the Ag ions with I ions to form AgI, thereby reducing metallic Ag formation (5–7). However, the effect of this combination of SDF and KI (SDF+KI) on remineralization, and on preventing further demineralization, has not been well researched. Therefore, the aims of this study were to investigate and compare the effects of SDF with SDF+KI on primary enamel and primary dentin lesions, under re- and de-mineralization conditions *in vitro,* using x-ray microtomography (XMT) and back scattered scanning electron microscopy (BSE-SEM).

## 2 Materials and methods

**Figure 1:**
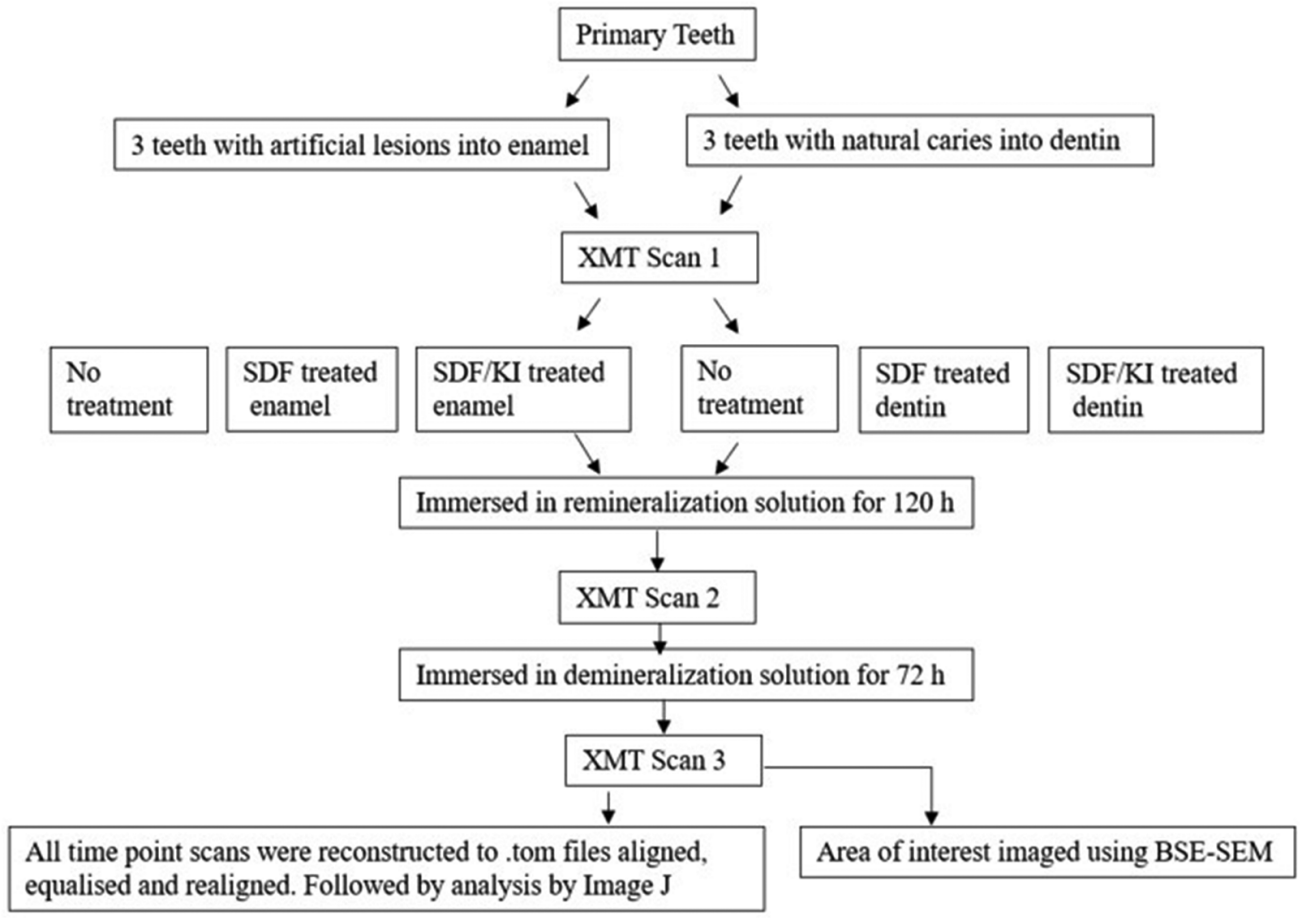
Schematic representation of the experimental method.

Six primary teeth were selected from the human tooth tissue bank at Queen Mary University of London (Ethics number- QMREC 0014/17). Three teeth with sound enamel, and three other teeth with natural dentin caries were selected for the experiment. Each sound enamel tooth was coated with nail varnish, leaving a 3 × 4 mm window and immersed in 50 ml demineralization solution (0.1M acetic acid buffered with sodium acetate at pH 4.5) for 72 h (8) in order to create an artificial enamel lesion. Both the enamel and dentin groups were divided into 3 subgroups: EC – Enamel Control; ES – Enamel with SDF application; ESK – Enamel with SDF followed by KI application; DC – Dentin Control; DS – Dentin with SDF application; DSK – Dentin with SDF followed by KI application. Each tooth had XMT scans using MUCAT2 (QMUL) at 90 keV and 180 µA for 18 h, at 3 timepoints: (1) Pretreatment; (2) after immersion in remineralization solution (2 mM CaCl_2_, 1.2 mM KH_2_PO_4_, 150 mM NaCl) for 120 h, with/without SDF (Riva Star), or SDF+KI (Riva Star) treatment; (3) after immersion in demineralization solution (as above) for 72 h (Figure 1). After image reconstructions, the three XMT images of the same sample in 3 timepoints were aligned, and their linear attenuation coefficients (LACs) were normalised to 40 KeV using in-house software so that they could be compared and analysed quantitatively. After the XMT scans, all teeth were embedded and sectioned. The region of interest was then investigated microscopically using BSE-SEM (9).

## 3 Results

### 3.1 Artificial enamel carious lesion

**Figure 2:**
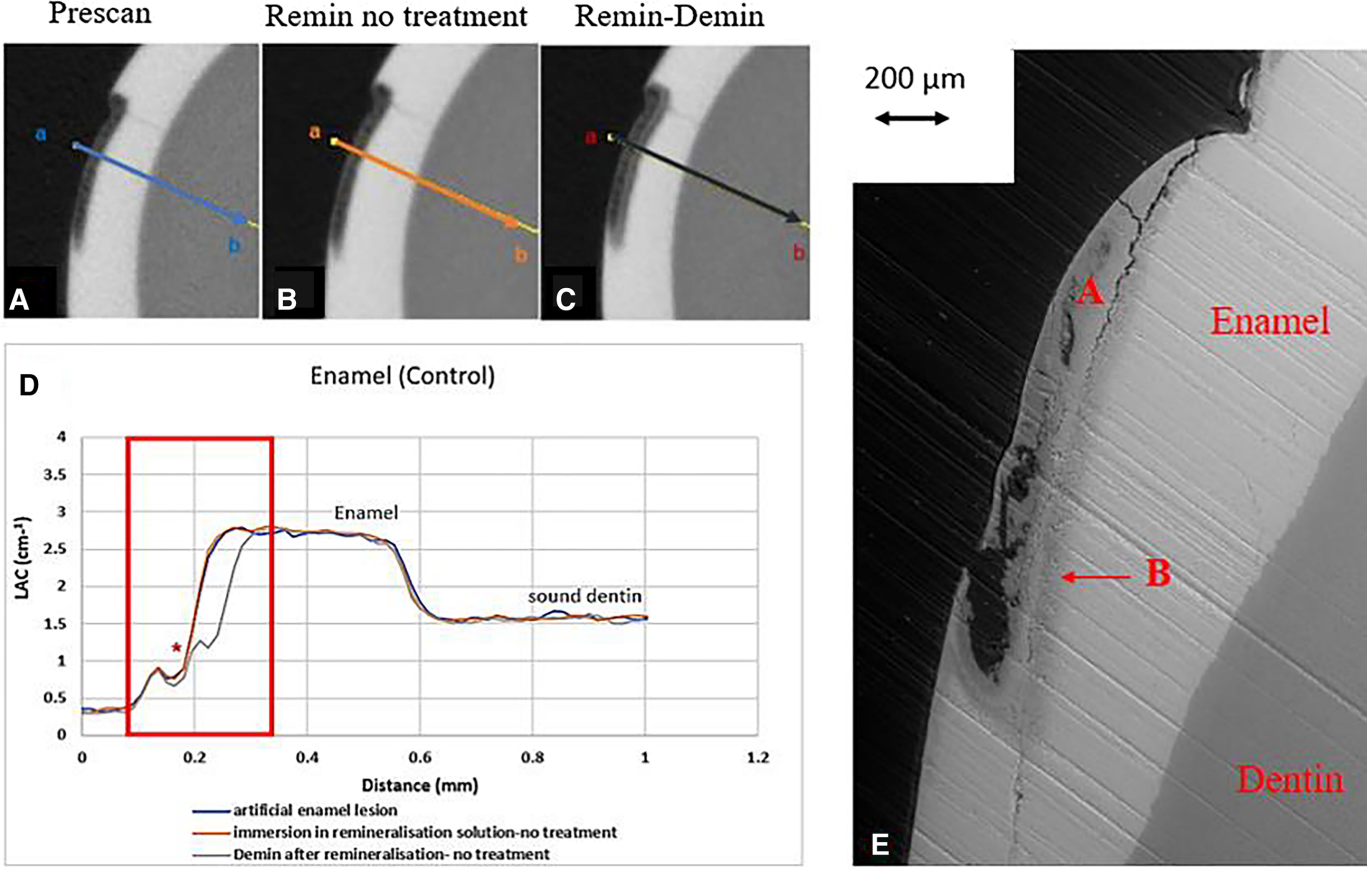
XMT slices of artificially created lesions in enamel with no SDF treatment showing no protection from demineralization in this control tooth. **(A)** Pretreatment scan; **(B)** after immersion in remineralization solution for 120 h; **(C)** subsequent immersion in demineralisation solution for 72 h; **(D)** line profiles of LACs for the 3 scans. **(E)** BSE-SEM image of the lesion in enamel: A- indicates the position of initial layer of demineralisation. B – extended demineralized region after subsequent immersion in demineralization solution.

**Figure 3:**
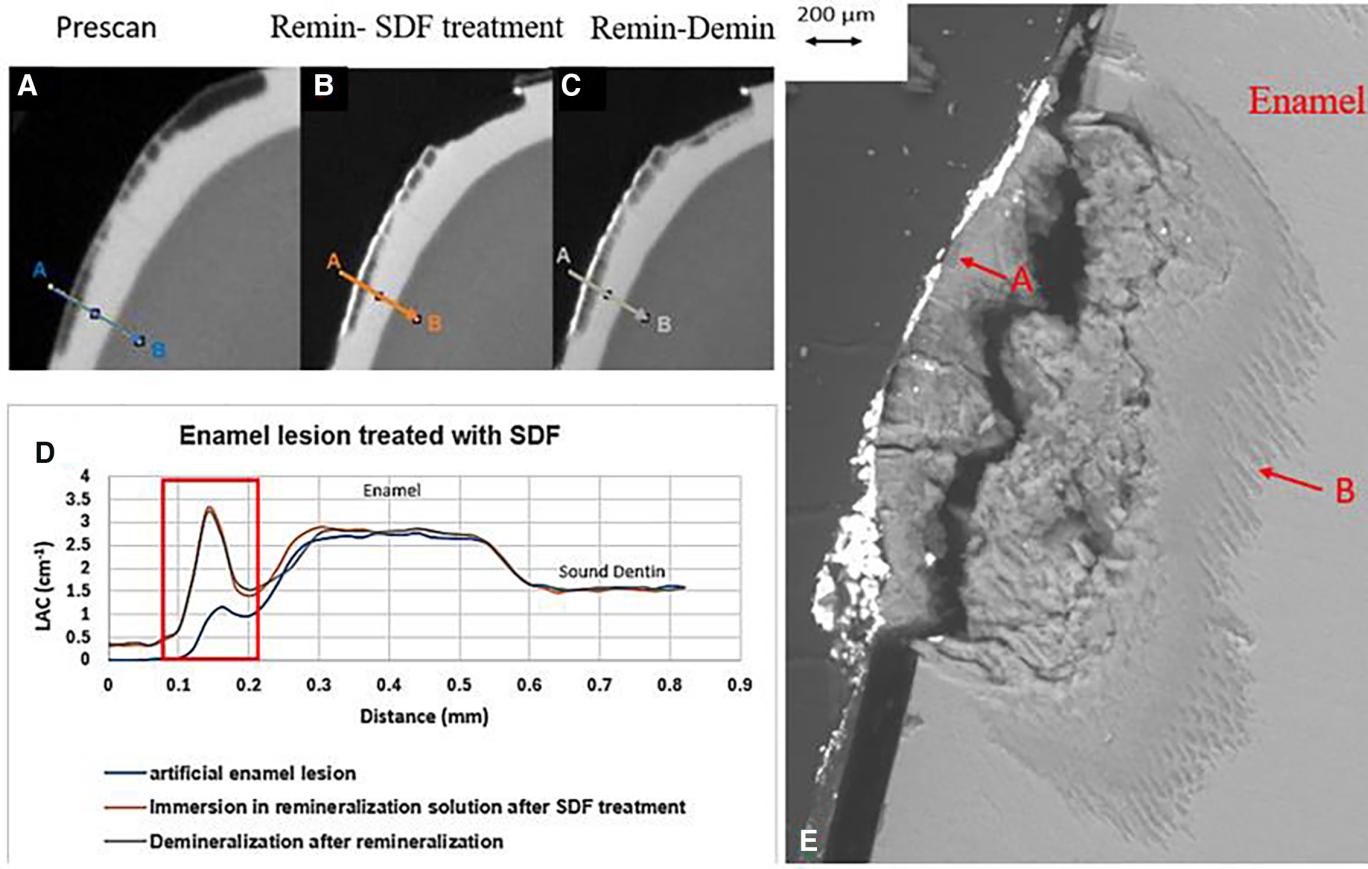
XMT slices of artificially created lesions in enamel with SDF treatment showing barrier formation on enamel surface with some penetration of SDF in porous enamel. **(A)** Pretreatment scan; **(B)** after SDF application and immersion in remineralization for 120 h; **(C)** subsequent immersion in demineralisation solution for 72 h; **(D)** line profiles of LACs for the 3 scans. **(E)** BSE-SEM image of the lesion in enamel: A- indicates the position of initial layer of demineralization; B – extended demineralized region.

**Figure 4:**
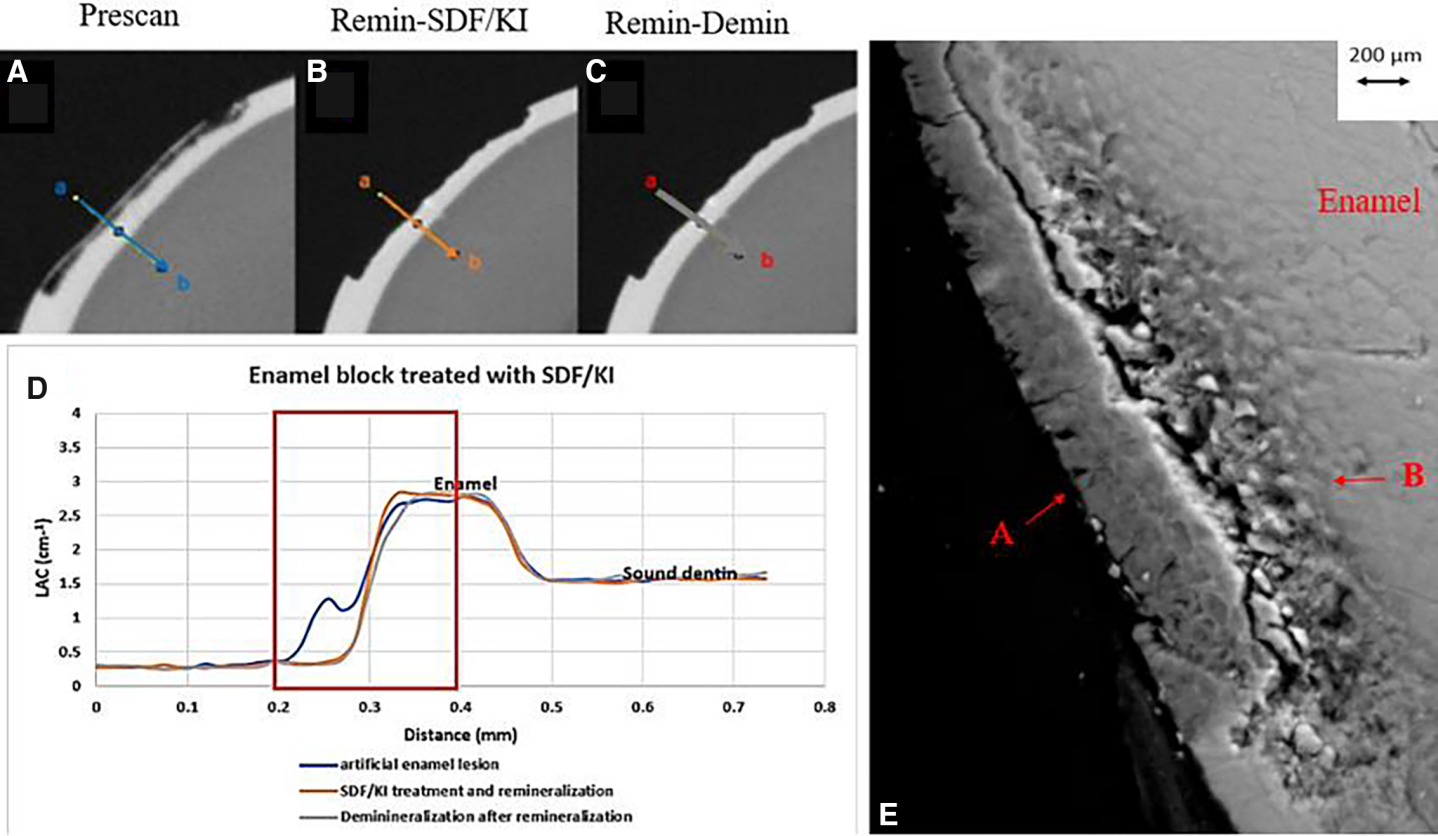
XMT slices of artificially created lesions in enamel with SDF+KI treatment showing no barrier formation and protection from demineralization. **(A)** Pretreatment scan; **(B)** after SDF+KI application and immersion in remineralization solution for 120 h; **(C)** subsequent immersion in demineralisation solution for 72 h; **(D)** line profiles of LACs for the 3 scans. **(E)** BSE-SEM image of the lesion in enamel: A- indicates the position of initial layer of demineralization B - extended demineralized region.

All the pre-treatment scans of the artificial demineralized enamel lesions show that each tooth had a subsurface demineralized lesion with a surface mineralized layer (Figures 2A,D, 3A,D, and 4A,D). With no SDF treatment, the lesion did not have any observable changes in LAC after immersion in remineralization solution for 120 h (Figures 2B,D). After subsequent demineralization for 72 h, the subsurface lesion extended further into enamel by about 0.06 mm (Figure 2C) and the line profile shows a second mineralized layer on the surface of the advancing front (Figure 2D). This feature could also be seen in the SEM image, which shows substantial loss of mineral (Figure 2E). With the application of SDF, there was a layer of high LAC (∼3.2 cm^−1^) material on the demineralized enamel surface, and into the lesion after remineralization (Figures 3B,D). After subsequent demineralization, the line profile (Figures 3C,D) shows that there was some loss of mineral in the depth of the lesion (indicated by the grey line shift to the right of the orange line). The radiopaque layer could be seen clearly in the BSE-SEM image covering the enamel, but it did not completely seal the surface. Therefore, a secondary demineralized layer could be detected (B in Figure 3E). With the application of SDF+KI, the initial surface mineralized layer was lost after remineralization (Figures 4B,D) and the normal enamel LAC was increased slightly from 2.6 to 2.8 cm^−1^. After subsequent demineralization, there was a small loss of mineral in sound enamel (∼0.02 mm).

### 3.2 Natural dentin carious lesion

**Figure 5:**
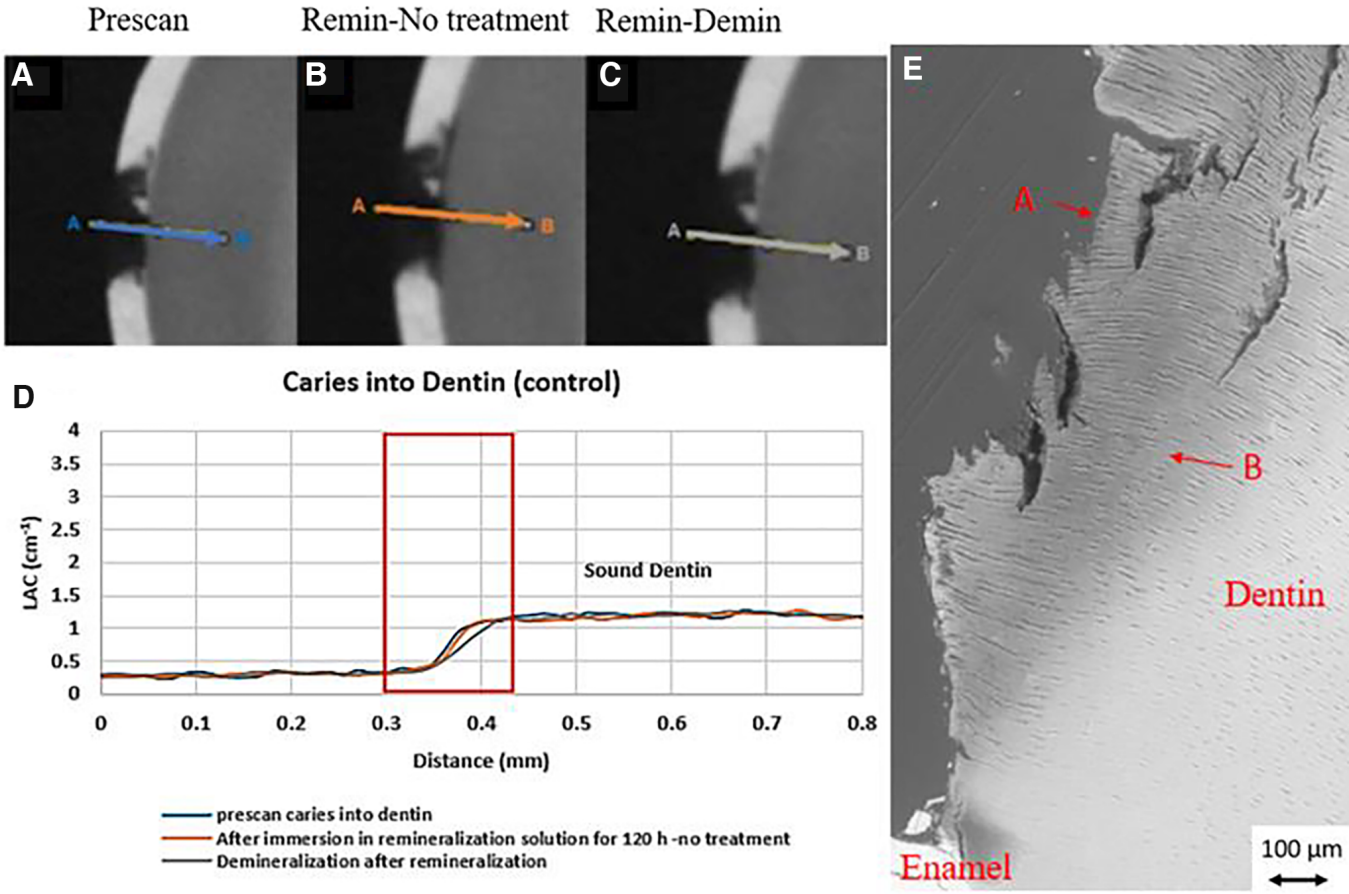
XMT slices of natural lesions in dentin with no SDF treatment showing no protection from demineralization in this control tooth. **(A)** Pretreatment scan; **(B)** after immersion in remineralization solution for 120 h; **(C)** subsequent immersion in demineralisation solution for 72 h; **(D)** line profiles of LACs for the 3 scans. **(E)** BSE-SEM image of the lesion in dentin showing dentinal tubules: A- indicates the position of initial layer of demineralization B – extended demineralized region in dentin.

**Figure 6:**
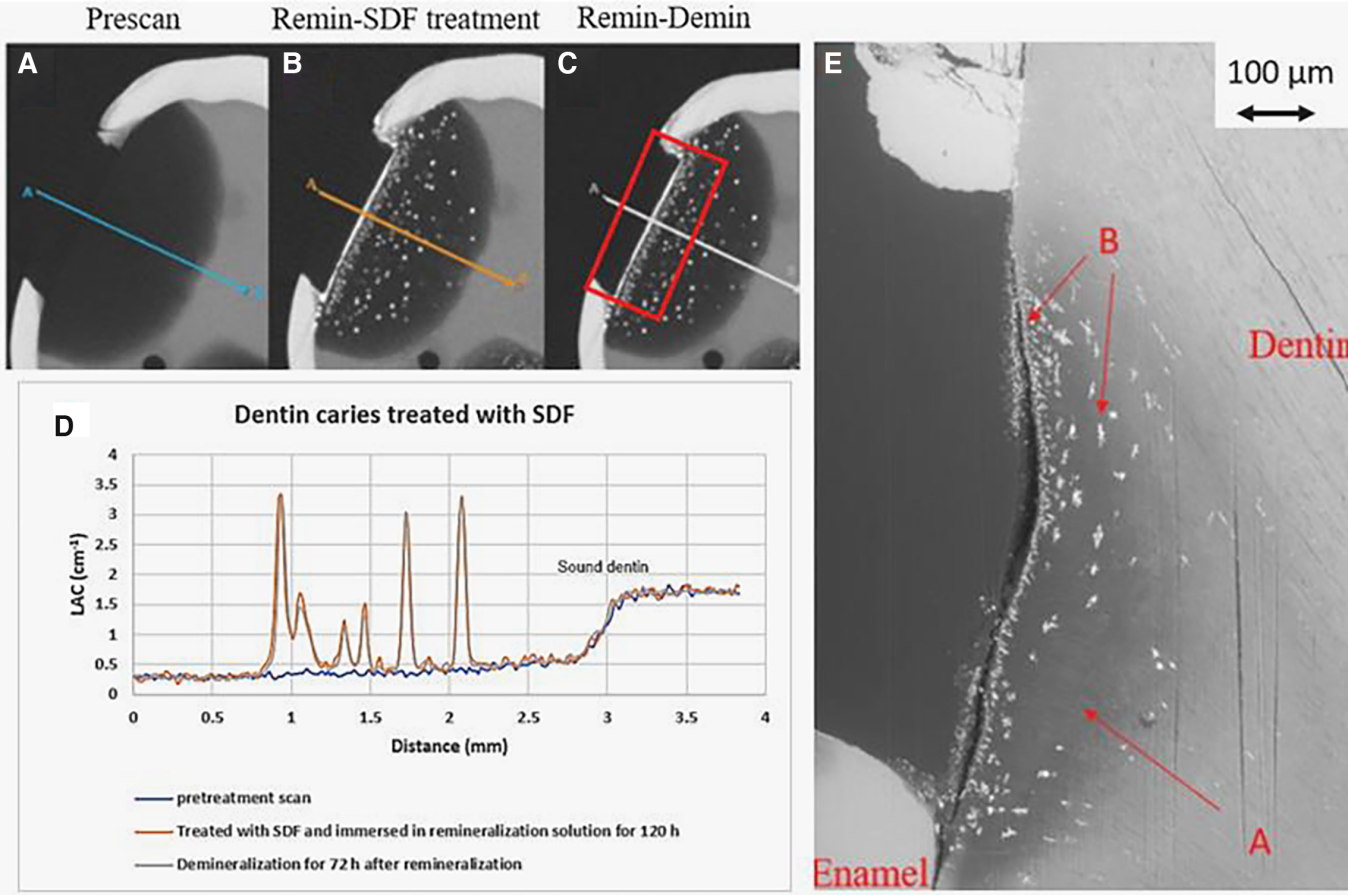
XMT slices of natural lesions in dentin with SDF treatment showing superficial radiopaque layer and Ag particles deposition in carious dentin matrix, protecting dentin from further demineralization. **(A)** Pretreatment scan; **(B)** after immersion in remineralization solution for 120 h; **(C)** subsequent immersion in demineralisation solution for 72 h; **(D)** line profiles of LACs for the three scans. The spikes indicate Ag deposition. **(E)** BSE-SEM image of the lesion in dentin showing no dentinal tubules: A - initial demineralized lesion; B - Ag particles in carious dentin matrix.

**Figure 7:**
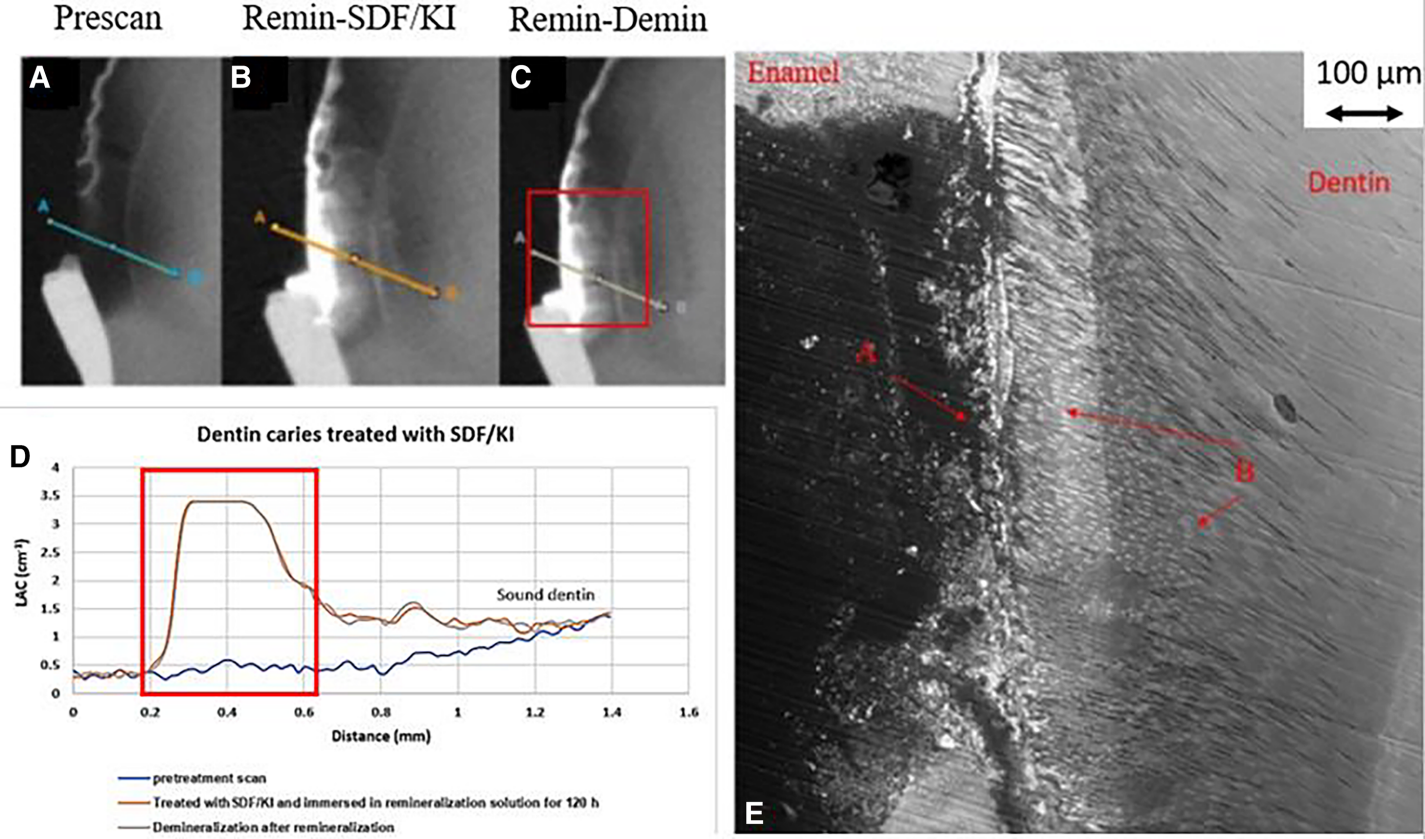
XMT slices of natural lesions in dentine with SDF+KI treatment showing thick superficial radiopaque layer and Ag particles deposition in carious dentin matrix, protecting dentin from further demineralization. **(A)** Pretreatment scan; **(B)** after immersion in remineralization solution for 120 h; **(C)** subsequent immersion in demineralisation solution for 72 h; **(D)** line profiles of LACs for the 3 scans. The high LAC layer are AgI. **(E)** BSE-SEM image of the lesion in dentin showing dentinal tubule structure: A- initial demineralized lesion with superficial Ag particles in carious dentin matrix B- Ag particles in carious dentin matrix and subsurface dentinal tubules.

In all the natural dentin carious lesions, there was no surface mineralized layer as observed in the artificial enamel lesions (Figures 5A,D, 6A,D, 7A,D). In the control sample without SDF application, remineralization did not seem to have an effect (Figures 5B,D). After subsequent demineralization, there was further loss of minerals in the sound dentin (Figures 5C,D). This could also be observed in the BSE-SEM image (B in Figure 5E). With the application of SDF, the DS sample had high LAC at the surface, and a speckled appearance with high LAC islands after remineralization inside the lesion (Figures 6B,D). After subsequent demineralization, no further loss of mineral was observed within the lesion or into the sound dentin (Figures 6C,E). In the BSE-SEM image, high density particles were observed inside the lesion (Figure 6E). No dentinal tubule structures were observed inside the lesion, but was apparent in the sound dentin. With the application of SDF+KI, the DSK sample had a thick layer of radiopaque material (LAC > 3 cm^−1^) from the carious dentin surface into the into the depth of the lesion (Figures 7B,D), after remineralisation. Even in the deeper part of the lesion, the LAC was increased to around 1.25 cm^−1^, similar to that of sound dentin. After subsequent demineralization, there was no observable change of LACs in the lesion (Figures 7C,D). The BSE-SEM image (Figure 7E) showed that the lesion maintained some dentinal tubular structure and the high-density materials were deposited along the direction of the dentinal tubules.

## 4 Discussion

### 4.1 Enamel carious lesions

Artificial enamel lesions were used because it is difficult to standardise natural enamel carious lesions. Also, in natural early enamel carious lesion, there is usually a surface mineralised layer, possibly with high fluoride content that may prevent penetration of SDF into the lesion. It is confirmed in this study that even with the thinner surface mineralized layer, the penetration of the SDF was limited on the surface (Figure 2B). As the outer surface layer was smooth, the Ag particles did not retain well. This was shown in the ESK sample (Figure 4) which showed total loss of the surface after SDF+KI remineralization (Figure 4B). This might be due to the surface layer was very thin and was lost during the application of SDF+KI.

In the enamel control sample (EC), no increase of LAC (*i.e*., no remineralization) was observed after immersion in remineralization solution for 120 h (Figure 2B). This might be due to the immersion time was too short; and/or the surface mineralized layer prevented any further exchange of ions in the deeper layer. However, when this tooth was re-immersed in demineralization solution for 72 h, there was further mineral loss in the body of the lesion, with an increase in depth (Figure 2C). It was noted the surface mineralized layer was intact and retained its LAC. Furthermore, there was a 2nd mineralized layer with the second wave of demineralization in the depth of enamel. This implies that clinically when enamel is subjected to cyclical de- and re-mineralization, the dynamics of ion exchange will create a complex enamel structure.

In the ES sample, after SDF application and remineralization, there was a very radiopaque layer on the enamel surface (Figure 3B). When SDF was in contact with the remineralization solution, AgCl is formed (4). From the high LAC value, this layer is due to the Ag content of AgCl. In this sample, the AgCl was retained, covering the surface of enamel. From the line profile (Figure 3D), the AgCl might have penetrated inside the lesion and increased LAC of the lesion. Although remineralization might also occur due to the high F content of SDF, the immersion time is too short for this to happen, as shown in the EC sample. Also, after demineralization, there is some mineral loss beyond the original lesion into normal enamel, showing that SDF does not prevent demineralization in enamel.

After application of SDF+KI and after remineralization, the surface layer of the ESK seemed to be destroyed (Figure 4B). As there was no acidic component in SDF+KI, the destruction was likely caused by the mechanical brushing during the application since the surface was thin and weak. However, there was a small increase in LAC in the normal enamel, indicating that a small amount of AgI (a product formed when SDF is mixed with KI) managed to be deposited on the surface and partly into the enamel. Once again, this layer did not protect the enamel from subsequent demineralization indicating no formation of acid resistant fluorapatite or fluoride substituted hydroxyapatite (FA/FSHA).

These results suggest that the SDF will penetrate into the enamel only if some porosities exist. Li et al. (10), reported the movement of silver ions through the pellicle along the prism boundaries. However, this current study shows that the penetration of SDF is limited to the surface of the demineralized lesion. In natural enamel carious lesion, the porous structure could be different from the artificial lesion. Clinically, when the lesion becomes black after SDF application, it indicates that the lesion is porous enough to retain the SDF byproduct that is photo reduced to metallic Ag, which might offer protection from bacterial invasion.

### 4.2 Dentin carious lesion

In natural dentin carious lesion (DC sample), no change of LAC was observed after immersion in remineralization solution for 120 h (Figure 5B). This implies that dentin caries is difficult to remineralize without the presence of fluoride. Hence, there was no protection when the tooth was subsequently immersed in demineralization solution for 72 h, resulting in a loss of mineral and decrease in LAC (Figure 5D). When SDF was applied to the dentin carious lesion (DS sample), after remineralization, a layer of high LAC (up to 3.4 cm^−3^ material was deposited on the surface of the lesion. This material was likely to be AgCl. Some of this material also penetrated into the lesion and randomly deposited in the dentin lesion. After subsequent demineralization, the LAC was unchanged. This implies that unlike enamel, FA or FSHA might have been formed inside the dentin lesions but was masked by the high LAC of precipitated AgCl. This might explain the multiple peaks in the line profile (Figure 6D). As FA/FSHA are more acid resistant and harder, subsequent demineralization did not result in further loss of mineral (11).

Several studies have reported occlusion of dentin tubules with the application of silver ions (2, 10, 12–15). Seto et al. (13) reported the formation of microwires of silver within the dentinal tubules but Kiesow et al. (14) and Menzel et al. (15) found particles of Ag occluding the dentinal tubules. However, if the dentinal tubule structure is destroyed, as shown in the BSE-SEM image (Figure 6E), Ag particles could only be retained within space in the matrix. This might explain the random high LAC island appearance in Figure 6B.

In the DSK sample which had SDF+KI application, there was a thick layer of high LAC material on the surface. Unlike the DS sample, the penetrated material had a structural pattern (Figure 7B). The BSE-SEM image (Figure 7E) showed that the dentinal tubule structure within the lesion was not destroyed. Hence, this radiopaque material, most likely to be AgI, was deposited inside the dentinal tubules, as shown by others (14, 15). Hence, it does not indicate that SDF+KI application results in deeper penetration than SDF, it depends on the structure of the natural dentin carious lesion. Similar to SDF, SDF+KI application would have formed FA/FSHA to harden the dentin and prevent mineral loss in subsequent demineralization (Figure 7D).

Regarding the remineralization effect of SDF, Heukamp et al. (16) found that SDF to be less effective than Cervitec F varnish on artificial enamel caries lesion, which agrees with the results of present study. |With regard to SDF on dentin, Cifuentes-Jiménez et al. (3) found that SDF and NaF resulted in an increase in mineral content, the formation of a crystalline precipitate with high flexural strength. They found no effect of acid challenge on dentin blocks treated with SDF under pH cycling. This agrees with this current study which shows application of SDF with or without KI protects dentin from acid attack.

## 5 Conclusion

The study presented provides valuable insights into the remineralization potential of SDF and its KI adjunct on both enamel and dentin lesions in primary teeth. The findings from this *in vitro* study shows that silver ions penetrate the enamel structure only in the presence of porosities, suggesting that SDF treatment may benefit sound enamel to prevent demineralization. The SDF application is most effect in carious dentin where Ag containing material is deposited on the surface and inside dentin tubule, with F containing apatite formation. This can then protect the dentin from acidic challenge. The adjunct of KI, used for reducing formation of black metallic silver, does not seem to hamper its protective property.

## Data Availability

The original contributions presented in the study are included in the article/Supplementary Material, further inquiries can be directed to the corresponding author.
